# Supplementary material for: The Construction and Exploration of a Comprehensive MicroRNA Centered Regulatory Network in Foxtail Millet (Setaria italica L.)
Source: Front Plant Sci. 2022 May 6;13:848474. doi: 10.3389/fpls.2022.848474 (PMC9121102; doi:10.3389/fpls.2022.848474)
Supplement: Supplementary file 1 [file Data_Sheet_1.zip › Sit-miR394_Seita.9G125200.1_4559_TPlot.pdf]

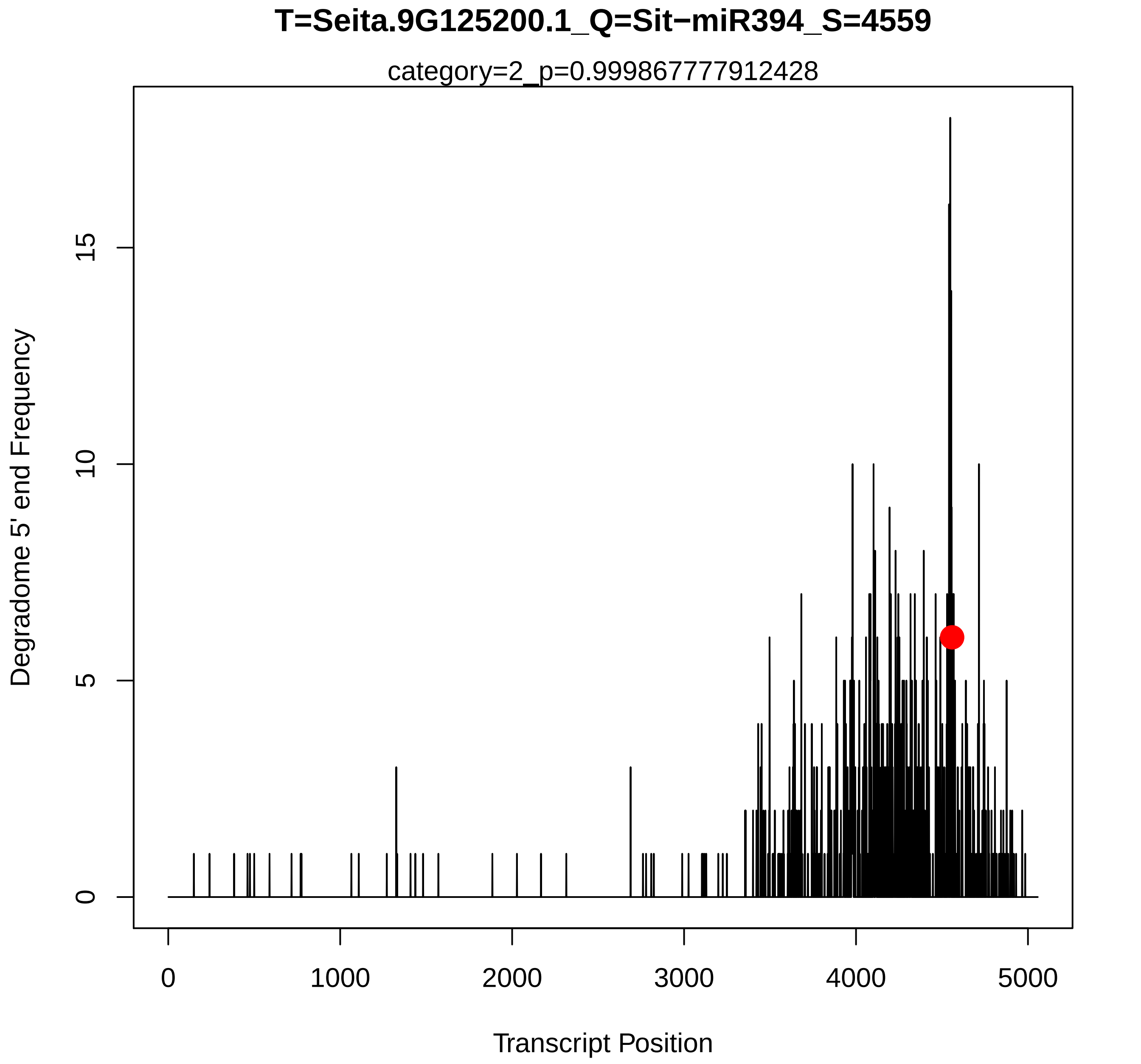

T=Seita.9G125200.1_Q=Sit−miR394_S=4559
category=2_p=0.999867777912428
Degradome 5' end Frequency
0
5
10
15
0
1000
2000
3000
4000
5000
Transcript Position
